# In Vivo and In Vitro Analysis of Age-Associated Changes and Somatic Cellular Senescence in Renal Epithelial Cells

**DOI:** 10.1371/journal.pone.0088071

**Published:** 2014-02-04

**Authors:** Birgit Berkenkamp, Nathan Susnik, Arpita Baisantry, Inna Kuznetsova, Christoph Jacobi, Inga Sörensen-Zender, Verena Broecker, Hermann Haller, Anette Melk, Roland Schmitt

**Affiliations:** 1 Department of Pediatric Nephrology and Gastroenterology, Medical School Hannover, Hannover, Lower Saxony, Germany; 2 Department of Nephrology and Hypertension, Medical School Hannover, Hannover, Lower Saxony, Germany; 3 Department of Pathology, Medical School Hannover, Hannover, Lower Saxony, Germany; University of Washington, United States of America

## Abstract

Acute kidney injury is a major clinical problem and advanced age is associated with ineffective renal regeneration and poor functional outcome. Data from kidney injury models suggest that a loss of tubular epithelial proliferation contributes to a decrease in renal repair capacity with aging, but aging can also lead to a higher severity of inflammation and damage which may influence repair. In this study we tested intrinsic age-dependent changes in tubular epithelial proliferation in young and old mice, by injecting low-dose lead acetate as a non-injurious mitogen. In parallel, we explored *in vitro* techniques of studying cellular senescence in primary tubular epithelial cells (PTEC). Lead acetate induced tubular epithelial proliferation at a significantly higher rate in young as compared to old mice. Old kidneys showed significantly more senescence as demonstrated by increased p16*^INK4a^*, senescence associated β-galactosidase, and γH2AX^+^/Ki-67^−^ cells. This was paralleled in old kidneys by a higher number of Cyclin D1 positive tubular cells. This finding was corroborated by a positive correlation between Cyclin D1 positivity and age in human renal biopsies. When tubular cells were isolated from mouse kidneys they rapidly lost their age-associated differences under culture conditions. However, senescence was readily induced in PTEC by γ-irradiation representing a future model for study of cellular senescence in the renal epithelium. Together, our data indicate that the tubular epithelium of aged kidney has an intrinsically reduced proliferative capacity probably due to a higher load of senescent cells. Moreover, stress induced models of cellular senescence are preferable for study of the renal epithelium *in vitro*. Finally, the positive correlation of Cyclin D1 with age and cellular senescence in PTEC needs further evaluation as to a functional role of renal epithelial aging.

## Introduction

Renal aging is associated with an increased susceptibility to acute stress and tubular cell injury. While the young kidney has a remarkable capacity to recover from acute injury, the aging kidney loses this repair reserve and instead develops an increasing tendency for tubular atrophy and interstitial fibrosis. Our previous data suggest that a loss in tubular epithelial proliferative reserve contributes importantly to inappropriate repair in the aged kidney [1], [2]. Under baseline conditions the renal tubular epithelium has a low rate of cellular turnover when compared to other tissues. In mouse kidney less than 1% of proximal tubular cells express proliferation markers under normal conditions [3], [4]. In response to acute damage, however, the renal epithelium can initiate a burst of proliferation which serves to repopulate and restore injured tubules [5]. This injury-response may lead to full functional recovery even after extensive tubule denudation. We have previously shown that the proliferative potential of tubular cells declines with chronological age [2], [4], [6]. In previous studies we linked the inability to increase cell cycling to somatic cellular senescence (SCS) by demonstrating that genetic induction of telomere shortening, as a model of telomere dependent SCS in mice, was associated with a decline in the tubular proliferative capacity [7]. Ablation of the pro-senescent p16*^INK4A^*, on the other hand, resulted in improved regeneration and better proliferation following acute ischemic renal injury [1].

SCS was initially described as an *in vitro* phenomenon in human fibroblasts that proliferate only for a finite number of cell passages before going into a G1 phase arrest [8]. When this stage is reached, the cells remain viable and metabolically active, but they irreversibly cease to replicate. There are two main pathways of SCS induction: replicative senescence and stress- and aberrant signaling-induced senescence (STASIS) [8]. Replicative senescence is caused by telomere shortening and dysfunction while STASIS is caused by extrinsic stresses that activate the p16*^INK4A^*-pRb pathway. In recent years, evidence for the role of SCS in renal aging and disease has accumulated [9], [10]. SCS has not only been described as part of the renal aging process [4], [6], [11], [12] but also seems to be a consequence of acute and chronic kidney damage as observed in hypertension, renal transplantation, glomerular disease and diabetic nephropathy [4], [13]–[15].

While a growing number of reports support the general link between SCS, defective proliferation and age-associated regenerative dysfunction, this issue has been difficult to address in renal studies in which proliferation is always induced by acute kidney injury [1], [2], [4], [7]. As acute kidney injury causes more damage in aged animals [1], [4], [16], [17] differences in proliferation were difficult to interpret and may have been due to differences in the damage load.

It was the goal of the present study to analyze age-dependent proliferative changes in a model that is not biased by these potential differences. To this end we analyzed differences in renal epithelial cell proliferation after short term exposure to low-dose lead acetate, which has previously been used as a non-toxic tubular mitogen [18]–[21]. In parallel we *s*tudied differences in cell senescence markers and we analyzed age-dependent changes in Cyclin D1 expression. Cyclin D1 is a cell cycle protein that has been suggested as a marker for proliferative potential of G1 phase arrested tubular cells [21], [22]. With the goal of establishing a suitable system for studies of renal SCS *in vitro* we tested isolated primary tubular epithelial cells (PTEC) from old and young mice and the effects of γ-irradiation on PTEC.

## Results

### Lead acetate induces tubular epithelial cell proliferation without causing acute renal damage in vivo

**Figure 1:**
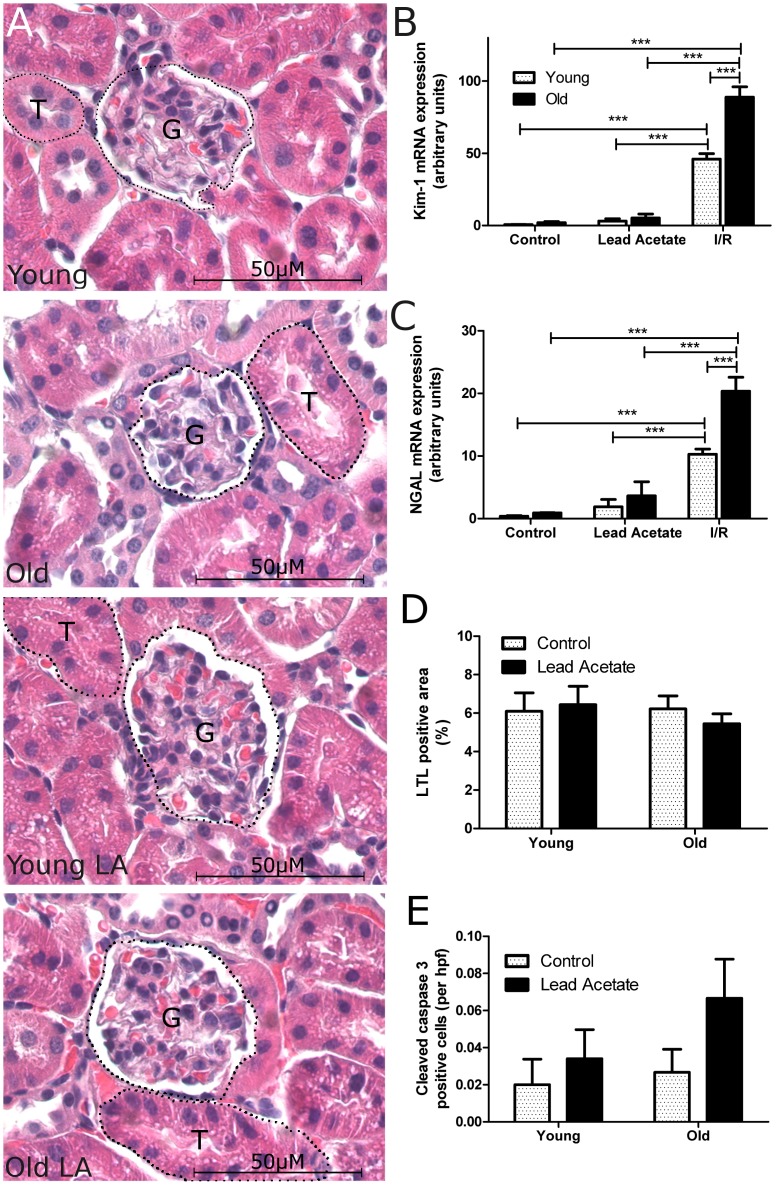
Administration of lead acetate does not cause damage to kidney tissue. Young and old mice were injected with 10/100 g body weight and sacrificed 36 hours later; alternatively mice underwent kidney ischemia/reperfusion injury by clamping of the renal pedicles and were sacrificed 24 hours thereafter. (A) Haemotoxylin-eosin staining of kidney sections from young and old mice with or without lead acetate treatment show no difference in renal microstructure (G represents glomerulus, T represents tubule); original magnification 400×. Quantitative PCR for damage markers (B) Kim-1 and (C) NGAL in control young and old mice as well as young and old mice exposed to lead acetate or after IR damage. (D) LTL damage score of young and old mice injected with lead acetate shows no difference in brush border damage. (E) Quantification of cleaved caspase 3 positive cells; n = 5, data are mean values ± SEM. ***P<0.001

Lead acetate has previously been described as a direct stimulus for renal tubular epithelial cell proliferation [19]. In contrast to other models that are used to investigate rapid tubular epithelial cell turnover such as ischemia/reperfusion or nephrotoxic injury [23], lead acetate acts as a universal mitogenic stimulus that does not cause cellular damage in short-term treatment [18]–[21]. In order to confirm these characteristics, and to exclude injurious effects, we first studied the impact of lead acetate on tubular cell integrity at 36 hrs after injection. Young adult and old (3–4 and 22–24 months) male C57Bl/6 mice were injected with 10 mg lead acetate/100 g body weight. Morphologically, we found no impact of lead acetate treatment on tubular epithelial microstructure when compared to control kidneys (Figure 1 A). Consistently, the expression levels of highly sensitive tubular injury markers NGAL and Kim-1 were unaltered after lead acetate exposure (Figure 1 B–C). This was in contrast to a dramatic up-regulation of NGAL and Kim-1 in young and old mice after ischemia/reperfusion injury (Figure 1 B–C). Moreover, there was no significant difference in lotus tetragonolobus lectin (LTL) damage scoring (Figure 1 D) or apoptosis in the kidney as measured by staining for cleaved caspase 3 (Figure 1 E).

### Lead acetate induces more cell proliferation in young than in old kidneys in vivo

**Figure 2:**
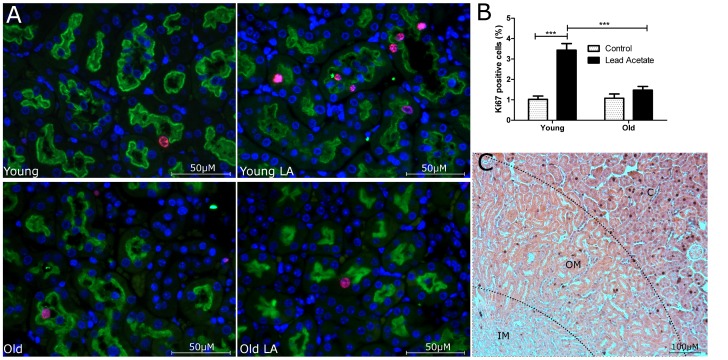
Lead acetate induces tubular cell proliferation in young but not old kidneys. Old and young mice were sacrificed 36/100 g body weight lead acetate. (A) Representative Ki-67 (red) and LTL (green) immunostaining of kidney sections from young and old mice with or without lead acetate treatment; original magnification 400×. (B) Quantification of Ki-67 positive cells; (C) representative immunostaining of Ki-67 showing the segments of the kidney (C represents cortex, OM outer medulla and IM inner medulla). n = 5, data are mean values ± SEM. ***P<0.001.

Kidneys from young and old mice were analysed at 36 hrs after lead acetate injection for proliferation of tubular epithelial cells using Ki-67 immunostaining. Consistent with previous reports [32] there was no discernable difference between young and old control kidneys which showed equally low numbers of proliferating tubular cells at baseline conditions (Figure 2 A). Lead acetate treatment however, induced a significant difference, caused by an increase in the percentage of proliferating tubular cells in young mice, but not in old mice (Figure 2 A–B). Cell proliferation was mostly found in proximal tubular segments showing costaining for brush border marker LTL (Figure 2 A). Accordingly, the majority of proliferating cells were located in cortex and outer medulla while very few Ki-67 positive cells were found in the inner medulla (Figure 2 C). Finally, there were no differences detected in phosphorylation or expression of MAPK signaling protein Erk p42/44 between the groups (data not shown), indicating that differences seen in proliferation were unlikely related to age-dependent changes in the MAPK signaling pathway.

### Baseline expression of cell cycle protein Cyclin D1 is higher in old kidneys than in young kidneys in vivo

**Figure 3:**
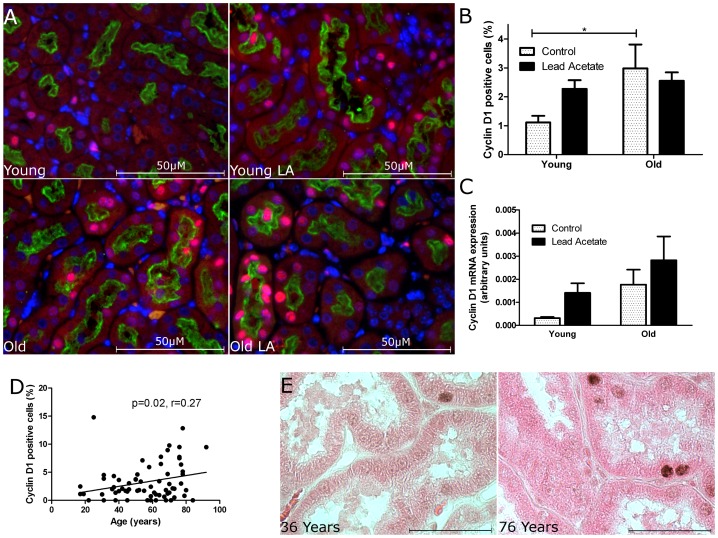
Baseline expression of cell cycle protein Cyclin D1 is higher in tubular cells of old kidneys than tubular cells of young kidneys. (A) Representative photographs of Cyclin D1 (red) immunostaining of kidney sections from young and old mice with or without lead acetate treatment. LTL (green) stains the brush border membrane of the proximal tubule; original magnification 400×. (B) Quantification of tubular cells with Cyclin D1 positive nuclei. (C) Analysis of Cyclin D1 mRNA expression. (D) Quantification of Cyclin D1 positive cells in renal transplant implantation biopsies (n = 36) and healthy renal tissue from nephrectomised patients (n = 22) shows a significant positive correlation between tubular Cyclin D1 expression and chronological age. (E) Representative photographs of Cyclin D1 immunostaining of kidney sections from a younger (36 years) and an older (76 years) human kidney; original magnification 400×. n = 5 for mice. Data are mean values ± SEM. *P<0.05.

To further analyze changes in cell cycling behaviour we measured the expression of Cyclin D1, a G1 Cyclin, which plays a key role in cell cycle regulation during the G1-S transition by cooperating with cyclin-dependent kinases [24]. Cyclin D1 was of particular interest, because it has been previously suggested that it characterizes G1 phase arrested tubular cells that are ready to start an immediate proliferative response if cell replacement is needed [21], [22]. According to this concept we had hypothesized, that younger kidneys might display more Cyclin D1 positive tubular cells since they show a faster proliferative response after acute damage [2] and after lead acetate stimulation. Surprisingly, we found more Cyclin D1 positive cells at baseline conditions in older kidneys as compared to young kidneys as shown by immunohistochemistry (Figure 3 A–B). In the great majority these cells were not cycling as evidenced by the lack of costaining with Ki-67 (not shown). The higher expression of Cyclin D1 in aged kidneys was corroborated by quantitative PCR revealing a trend for increased mRNA levels (Figure 3 C).

To test the relevance of Cyclin D1 for the human situation, we analyzed if there was an age-dependent effect on Cyclin D1 expression in human kidneys. Immunohistochemistry on healthy renal transplant implantation biopsies (n = 36) and healthy renal tissue parts from nephrectomised patients (n = 22) showed a significant positive age-correlation between tubular epithelial Cyclin D1 expression and chronological organ age (Figure 3 D–E). Taken together, these results indicate that the proposed role of Cyclin D1 as a marker of mitotic potential in tubular epithelial cells [21], [22] is not applicable in older individuals.

### Aged mouse kidneys show a higher load of senescent tubular cells in vivo

**Figure 4:**
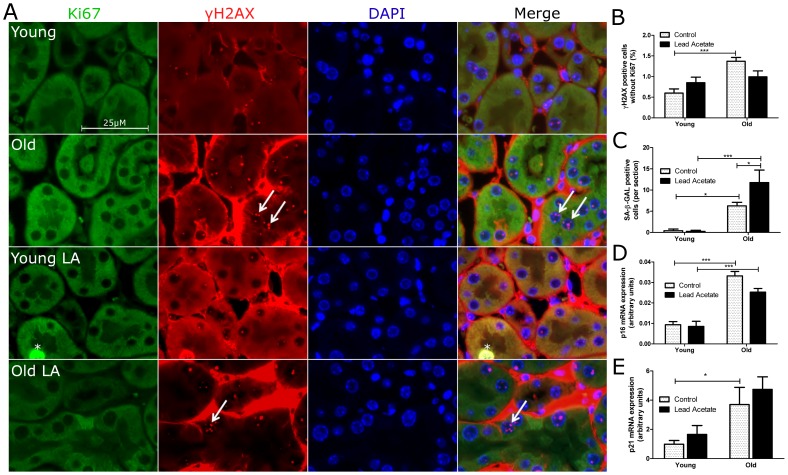
Lead acetate treatment does not alter senescence markers in cells in the kidneys of old mice. (A) Representative double immunostainings for phospho-γH2AX (red) and Ki-67 (green) in kidney sections from young and old mice with or without lead acetate treatment. Nuclei with more than 4 foci were considered positive (arrowheads) while double positive cells were not considered senescent (asterisk); red staining in the interstitial space is due to secondary antibody binding to native IgG; original magnification 400×. (B) Quantification of γH2AX positive and Ki-67 negative nuclei. (C) Quantification of SA-β-GAL positive cells. (D) Quantitative PCR for p16*^INK4a^*. (E) Quantitative PCR for p21. n = 5, Data are mean values ± SEM. *P<0.05; **P<0.01; ***P<0.001.

In order to define whether the lack of lead acetate induced epithelial proliferation in old mice was associated with a higher load of senescent cells we used different markers to identify SCS. First, we performed double immunostaining for Ki-67 and γH2AX, a marker combination considered highly sensitive for cellular senescence if four or more γH2AX foci are present in Ki-67 negative nuclei [1], [25]. Old control kidneys showed significantly more γH2AX^+^/Ki-67^−^ tubular cells (Figure 4 A–B). Similarly, old kidneys contained significantly more senescence associated-β-galactosidase (SA-β-GAL) positive tubular cells with a further increase after lead acetate treatment (Figure 4 C). Expression of p16*^INK4a^*, a cyclin-dependent kinase inhibitor and a biomarker for telomere independent senescence [6], was also significantly higher in aged kidneys (Figure 4 D). Moreover, p21, the transactivational target of p53 was expressed significantly more in kidneys of old animals (Figure 4 E). Together these data are consistent with a higher load of senescent tubular cells in aged mouse kidneys.

### In vitro culturing of primary tubular epithelial cells (PTEC) induces SCS-associated changes

Previous studies suggest a strong impact of the systemic milieu on age-dependent cell homeostasis and proliferation [26], [27]. To test whether *in vivo* differences in proliferation after lead acetate exposure were dependent on systemic factors, we conducted *in vitro* assays using freshly isolated cortical tubules (Day 0) and subsequent primary tubular epithelial cell (PTEC) cultures from young and old mice (Day 3 and Day 6).

**Figure 5:**
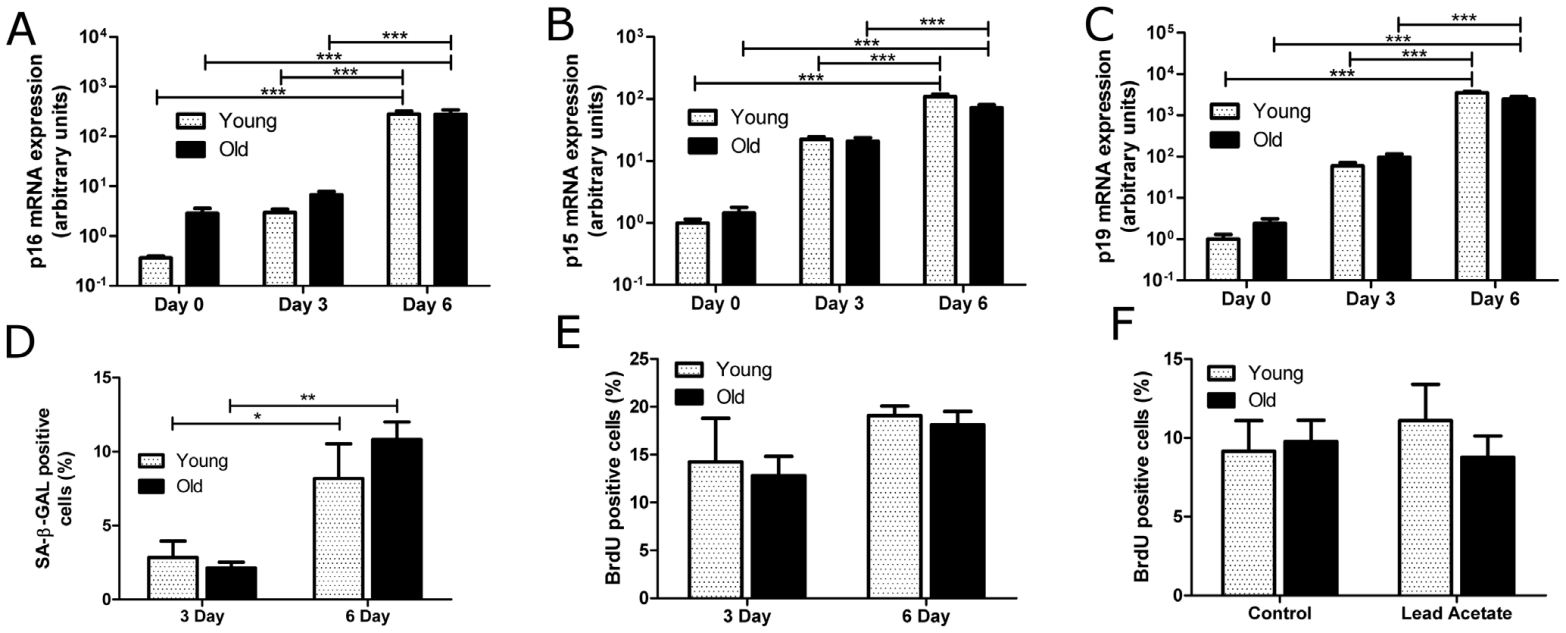
*In vitro* culturing of primary tubular epithelial cells (PTEC) induces SCS-associated changes. PTEC were isolated from young and old mice and harvested on day 0, day 3, or day 6 of culture. Quantitative PCR for (A) p16*^INK4a^* (B) p15*^INK4b^*, and (C) p19*^ARF^* in PTEC. (D) Quantification of SA-β-GAL staining on day 3 and 6 of PTEC culture. (E) Quantification of cells stained positive for BrdU on day 3 and 6 of PTEC culture. (F) BrdU uptake after lead acetate treatment in PTEC from young and old mice on day 6 of culture. n =  at least 4 separate mice, data are mean values ± SEM. *P<0.05; **P<0.01; ***P<0.001.

As anticipated, we found a trend for higher expression of senescence marker p16*^INK4a^* and cell cycle inhibitors p15*^INK4b^* and p19*^ARF^* in freshly isolated tubule preparations from old as compared to young kidneys (Figure 5 A–C). These differences between young and old were small as compared to the strong progressive up-regulation of p16*^INK4a^*, p15*^INK4b^* and p19*^ARF^* observed in PTEC of both age groups at 3 and 6 days of *in vitro* culture (Figure 5 A–C). The up-regulation was paralleled by a significant increase in SA-β-GAL positive cells from day 3 to day 6 in both age groups (Figure 5 D). Cell proliferation as measured by BrdU uptake showed similar baseline levels between young and old PTEC (Figure 5 E). Lead acetate exposure at 70% confluence resulted in no significant increase in proliferation in PTEC (Figure 5 F).

### **γ**-irradiation is a reliable method to induce SCS in PTEC in vitro

**Figure 6:**
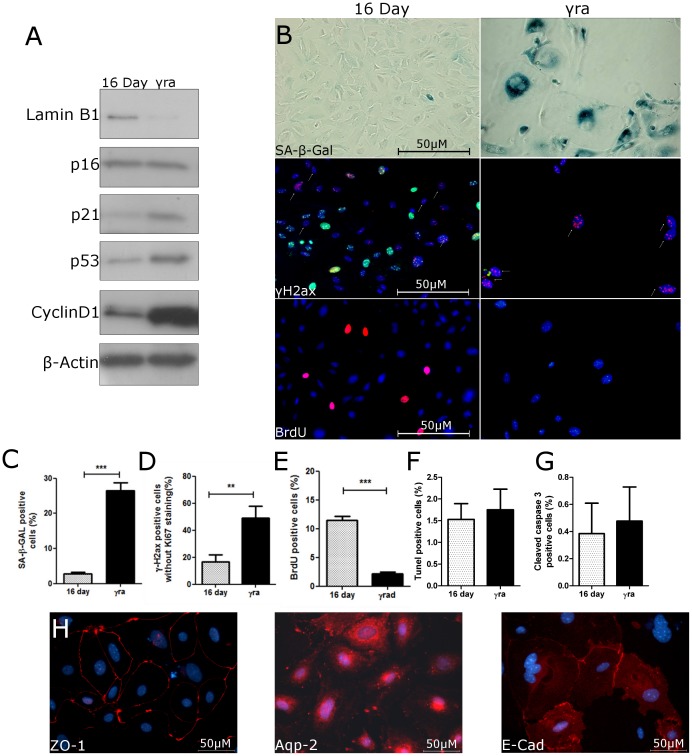
γ-irradiation induces senescence in PTEC and leads to increased Cyclin D1 expression. PTEC were isolated and grown for 6 days in culture before being exposed to 10 Gy γ-irradiation. After y-irradiation, cells were split and grown for 10 days and tested for senescence markers. Controls were grown for 6 days, split and grown for another 10 days. (A) Representative immunoblots for Lamin B1 and cell cycle regulators p16*^INK4a^*, p21, p53, and Cyclin D1. (B) Representative photographs of SA-β-Gal, γH2AX and BrdU; original magnification 400×. Quantification of (C) SA-β-Gal, (D) γH2AX^+^/Ki-67^−^, (E) BrdU, (F) TUNEL, and (G) cleaved caspase 3 positive cells in cultures of control and γ-irradiated PTEC. (H) Representative photographs of epithelial markers ZO-1, Aqp-2, and E-Cadherin in γ-irradiated PTEC; original magnification 400×. Data are mean values ± SEM. **P<0.01; ***P<0.001.

In order to establish a system in which the process of SCS could be experimentally induced, PTEC from young mice underwent 10 Gy of standardized γ-irradiation. 10 Gy, or more, of irradiation has been used as a standard tool to induce SCS in fibroblasts in many different studies [28]–[30]. To examine the pathway that leads to SCS after γ-irradiation, irradiated PTEC were compared with PTEC of the same day and passage. Immunoblot for Lamin B1 revealed far less expression in γ-irradiated PTEC indicating an increase in the amount of cells undergoing SCS [31] (Figure 6 A). Furthermore, cell cycle regulators p21 and p53 were both upregulated in γ-irradiated PTEC whereas p16*^INK4a^* expression showed no further increase. These data suggest that γ-irradiation causes p53 associated senescence while cell culture-stress induces upregulation of p16*^INK4a^* (Figure 6 A). Moreover, γ-irradiated PTEC had signs of typical senescent morphology including cell enlargement and flattening (Figure 6 B), higher amounts of SA-β-Gal and more γH2AX^+^/Ki-67^−^ cells (Figure 6 B–D). In parallel, proliferation was significantly decreased (Figure 6 E). γ-irradiated PTEC did not show a change in apoptosis as indicated by TUNEL staining and staining for cleaved caspase 3 (Figure 6 F–G), and also retained expression of markers found on renal epithelial cells such as ZO-1, Aqp-2, and E-Cadherin (Figure 6 H). Taken together, these data indicate that γ-irradiation is a reliable method to study SCS in PTEC.

## Discussion

After acute renal injury, tubular cells have a remarkable capacity to rapidly progress from quiescence to proliferation [3], [5]. Experimental models of renal regeneration indicate that this functional switch may be delayed or insufficient in aged rodents [2], [32]. However, previous studies were questioned because they relied on injury models that may induce different damage loads between young and old kidneys [2], [32]. We therefore challenged kidneys using lead acetate as a primary mitogen and found that the proliferative response was significantly diminished in aged tubular epithelium. The drop in proliferative potential was associated with a higher load of senescent cells. These age-associated characteristics of tubular epithelial cells are rapidly neutralized during cell culturing in which a rapid development of pro-senescent characteristics is dominating both age groups.

A major advantage of primary cultures is that the cells have not been modified and they are therefore thought to reflect the *in vivo* situation more closely than cell lines. This is a potential advantage for studies of aging in which immortalized cell lines might utilize molecular pathways that counteract normal aging and block the development of age-dependent programs such as SCS. In this respect, PTEC can be a suitable system to study features that renal tubular cells have acquired during aging [2], [32]. However, in our present study, PTEC quickly lost age-dependent differences under the impact of progressive SCS induction during *in vitro* culture conditions. Our data indicate that the strong induction of a pro-SCS phenotype in cultured PTEC overrides pre-existing more subtle changes of aged tubular cells that were acquired *in vivo*. Regardless of their age PTEC massively up-regulated cell cycle inhibitors p16*^INK4a^*, p15*^INK4b^* and p19*^ARF^* without exhibiting other typical features of a senescent phenotype. High expression levels of cell cycle inhibitors were accompanied by a stable proliferation rate and a normal morphology in PTEC at early time points. Mitogenic molecules in the cell culture medium signaling may provide stimulation strong enough to overcome the upregulation of negative cell cycle regulators attempting to arrest constant proliferation. This may be similar to ectopic expression of the oncoprotein *ras* in human primary fibroblasts where there is an induction of hyper-proliferation followed by premature senescence [33]. Furthermore, an artificial environment which includes the sudden lack of surrounding cell types and extracellular matrix components, together with abnormal concentrations of nutrients, growth factors and oxygen may lead to a ‘culture shock’ [34]. This ‘culture shock’ has been linked to stress-induced cell cycle arrest and senescence in different cell types [35]. According to our experience, results are subjected to variation in different laboratories because of small variables in culturing protocols such as the isolation method and the type of medium used. Our data make the usability of the PTEC system therefore questionable for studying age-associated differences that the cells have acquired *in vivo*. To date no unique biomarker is able to define a cell as senescent but senescent cells exhibit some characteristics like cell cylce arrest, SA-β-GAL activity, expression of cell cycle inhibitors, morphological transformation, Lamin B1 loss. [31], [35]. Standard irradiation provided a tool to establish a stable senescent PTEC system which fulfilled the above-named criteria and maintained epithelial features. We suggest that PTEC can be used to study SCS after γ-irradiation, which might be as valuable and reproducible for studying renal epithelial aging as it has been for the study of aging processes in other cell types [29], [36], [37].

Our data are consistent with the hypothesis that the regenerative loss of renal aging is associated with a higher load of SCS in tubular epithelial cells. This is also in line with our previous findings showing that interference with p16*^INK4a^* leads to better renal repair [1], [2]. A new important finding of our study was the age-associated increase in Cyclin D1 positive tubular epithelial cells in murine and human kidneys. Moreover γ-irradiation led to a robust increase of Cyclin D1 protein in senescent PTEC. Together with its partners CDK4 and CDK6, Cyclin D1 is a classic regulator of the G1 phase of the cell cycle by phosphorylation and inactivation of retinoblastoma protein (pRb) [24]. Overexpression of Cyclin D1 plays an important role in several cancers by driving mitotic processes [24]. Interestingly, it has previously been shown that depending on the context Cyclin D1 can also have inhibitory rather than stimulatory effects on cell cycling. In senescent fibroblasts, Cyclin D1 levels are significantly increased and experimental overexpression of Cyclin D1 can inhibit cell growth [38], [39]. An age-associated increase in epithelial Cyclin D1 has also previously been shown in rodent livers [40]. The reason for this Cyclin D1 increase in hepatocytes is unclear but it was speculated that it might reflect a disrupted cell cycle progression from the late G1 phase into the S phase [41]. A similar disturbance in cell cycling which might be linked to cell-cycle arrest could also be responsible for the age-related increase in tubular epithelial Cyclin D1. Leontieva et al recently linked the expression of cell cycle inhibitors p21 and p16*^INK4a^* and the growth promoting mTOR pathway to Cyclin D1 overexpression [42]. They showed that accumulation of Cyclin D1 can accompany the process of geroconversion, in which cell cycle inhibitors are induced in the presence of ongoing mTOR activity [42]. Since Cyclin D1 expression is already high in cells of old kidneys, further mitogenic stimuli may not increase the expression. Functionally it is not clear whether the increased baseline expression of Cyclin D1, observed in our studies, causes or is a consequence of cell cycle arrest. Our observations did not support the previous hypothesis that Cyclin D1 is labelling a tubular cell reserve that is ready to undergo rapid division if needed [21]. A better understanding of Cyclin D1 function and on its impact on renal cell cycle progression during the aging process might help to explain age-dependent differences in tubular epithelial cell biology.

A surprising finding with regard to the lead actetate model we used was the very moderate, but significant increase of the proliferative index. The effect of lead acetate on proliferation in rat kidneys had been much greater [20], [21]. The main goal of our analysis was to use lead acetate as a pro-mitotic substance without causing relevant cell damage, as damage could have functioned as a secondary pro-mitotic trigger. As we chose not to further increase the dose, we are unable to further explore dose-effect relationships of lead acetate and proliferation in our model. As all published studies were performed in rat models, it is conceivable that the difference is caused by interspecies differences. Another shortcoming of our model is that we cannot exclude differences in lead acetate distribution between young and old mice as we did not assess for lead acetate levels within renal tissue following injection.

In conclusion, our data show that tubular epithelial cells develop an intrinsic barrier to proliferation with advancing age. SCS is likely to play a role herein, but additional mechanisms of cell cycle inhibition, such as increased Cyclin D1, might also contribute. Methodologically, PTEC biology is highly influenced by ‘culture shock’ related stress, which causes age-dependent differences to vanish in culture. The *in vitro* SCS induction is thus largely independent of the age of the donor cells. Alternatively, γ-irradiation of PTEC might serve as a relevant and reproducible system for studying age and SCS dependent changes renal tubular cells.

## Methods

### Animals

Male C57BL/6 mice were housed and aged under standard conditions in Phenos GmbH animal laboratories (Hannover, Germany). Mice used for experimentation were either 3–5 months (young) or older than 18 months (old) old and weighed 28–35 g. All experimental procedures were in agreement with institutional and legislator regulations and approved by the Niedersächsisches Landesamt für Verbraucherschutz und Lebensmittelsicherheit.

### Ischemia/Reperfusion

Renal ischemia/reperfusion injury was induced in mice through unilateral pedicle clamping as previously described [43]. In short, mice were anesthetized with isoflurane, median laparatomy was performed, the left renal pedicel was dissected and a nontraumatic vascular clamp was applied for 27 minutes. Mice were sacrificed 24 hours after the operation and kidneys were harvested and flash frozen in liquid nitrogen for mRNA, protein or cryosection analysis, or fixed in 4% PFA for paraffin section analysis.

### Lead Acetate injections

Mice from both groups were injected i.v. through the tail vein with either 10 mg/100 g body weight lead acetate (Thermo Fischer Scientific, Schwerte, Germany) or with vehicle only. Mice were sacrificed 36 hours after injection kidneys were harvested and representative sections were either flash frozen in liquid nitrogen for mRNA, protein or cryosection analysis, or fixed in 4% PFA for paraffin section analysis.

### PTEC and BrdU uptake

Primary tubular epithelial cells (PTEC) were isolated as previously described [2]. Mice were anesthesized with isoflurane and kidneys were harvested after cardial perfusion with Hanks 199 medium (Promo Cell, Heidelberg, Germany) with 0,125% collagenase. Kidneys were digested at 37°C in a bubble-agitated collagenase solution for 40 minutes and tubules were then separated by size in a 40 µM cell strainer (BD, San Jose, USA) and plated with Renal epithelial cell medium I or II (PromoCell). For day 0 time point, cells were lysed by cell shredder in RLT buffer (Qiagen, Hilden, Germany), and mRNA was isolated by RNeasy kit according to manufacturer's protocol (Qiagen). Tubules were allowed to attach and grow in cell culture at 37°C and 5% CO_2_. Cells were harvested on day 3 and 6. Day 6 PTEC cultures were irradiated with 10 Gy (for 4∶51 minutes) of γ-irradiation. Cells were passaged the following day in a split ratio of 1∶4. Experiments were performed 10 days after y-irradiation. 16 day control samples were split to the ratio of 1∶4 on day six and experiments were performed 10 days thereafter. For BrdU uptake experiments PTEC from young and old mice were stimulated with 50 µM lead acetate for 24 hours after 6 days of culture. PTEC were exposed to 20 µM BrdU (Roche, Basal, Switzerland) and incubated for 4 hours under standard conditions. PTEC were fixed, permeabilized and flourescently stained for BrdU with Anti-BrdU antibody (Sigma, St. Louis USA). Alternatively 5-Bromo-2′-deoxy-uridine Labeling and Detection Kit I (Roche) was used according to manufacture's protocol with a 2 hour incubation time.

### Immunostaining

Immunostaining was performed on 4 µm sections from formalin fixed paraffin embedded tissues using the following primary antibodies: anti-Cyclin D1 (Thermo Fisher Scientific, SP4), rabbit anti-Ki-67 (Thermo Fisher Scientific, SP6), anti-Ki-67 (Dako, Glostrup, DK, MIB-5), anti-γH2AX (EMD Millipore, Billerica, USA, JBW301), polyclonal rabbit anti-cleaved caspase 3 (Cell Signaling, Danvers, USA, 5A1E) Ki67 and Cyclin D1 stains in mice were blocked with Universal Blocking Reagent (BioGenex, San Ramon, US) and visualized using the Envision monoclonal DAB system (Dako) or fluorescent secondary antibody Alexa Fluor 488 donkey anti-mouse IgG (Invitrogen, Carlsbad, US). Human material for Cyclin D1 immunohistochemistry comprised tumor-free tissue sections from nephrectomies of patients with renal carcinoma and renal transplant implantation biopsies (Department of Pathology, Medical School Hannover) all samples were archived samples approved by the Hannover Medical School ethics commission under human implantation biopsy vote 5346 and all patients gave written informed consent for the medical procedure from which the material was derived. Quantification of Cyclin D1 and Ki-67 expressing cells was done by counting of positive cells in 10 randomly chosen, non-overlapping fields (x400 (human: x200) magnification) in cortex. For Ki67 γH2AX double stains the fluorescent secondary antibodies, namely anti-rabbit Alexa Fluor 488 and anti-mouse Alexa Fluor 555 (Invitrogen), were used for visualization in the presence of a DAPI counterstain. Tubular cells negative for Ki-67 and containing more than 4 positive γH2AX foci per nucleus were counted as senescent cells [1]. The percentage of senescent cells was quantified by counting positive cells in 15 randomly chosen, non-overlapping HPFs (X400). For cleaved caspase 3 immunohistochemistry sections were blocked with 5% non-fat milk, visualized using the Envision monoclonal DAB system (Dako) and quantified by counting the average of positive cells/HPF in 30 randomly chosen, non-overlapping HPFs (200 x). For Cyclin D1 immunofluorescence on PTEC, cells were fixed in 4% PFA, blocked and permeabilized with PBS containing fetal calf serum, fish gelatine, BSA and Triton X-100. Anti-Cyclin D1 (Santa Cruz Biotechnology, Santa Cruz, US, DSC-6) was applied for 1 hr at room temperature followed by the visualization with the fluorescent secondary antibody Alexa Fluor 555 (Invitrogen) and counterstain with DAPI. Quantification of Cyclin D1 expressing cells was done by counting of positive cells in 10 randomly chosen, non-overlapping fields (x200) magnification). For ZO-1, E-Cadherin and Aqp-2 stainings, PTEC were fixed with 4% PFA and permeabilized with Triton X-100. Cells were blocked and incubated with primary antibodies: anti-ZO-1 (Invitrogen, 40-2200), anti-E-Cadherin (Cell Signaling, 24E10) and anti-Aqp-2 (Abcam, ab85876). Cells were washed and incubated with subsequent secondary antibody, washed again, and mounted with DAPI containing mounting medium. For TUNEL staining on PTEC the *In situ* Cell Death Detection Kit Fluorescein (Roche) was used according to the manufacturer's protocol.

#### LTL Damage Scoring

For histochemial assessment of tubular damage we used fluorescein labeled lotus tetragonolobus lectin (LTL, Vector Laboratories, Burlingame, USA), which identifies proximal tubules with an intact brush border. The quantitative assessment (LTL damage score) of positive tubules was done by analyzing total surface area and intensity of staining in 10, 200× random fields per kidney using ImageJ software (NIH).

### Senescence-associated-β-galactosidase staining

Senescence-associated-β-galactosidase (SA-β-GAL) staining on cultured cells was performed as described [44]. Briefly, the cells were washed with PBS and fixed with 2% formaldehyde/0.2% glutaraldehyde in PBS at room temperature. The cells were rinsed with PBS and incubated over night at 37°C with the X-GAL staining solution pH 6 (40 mM citric acid/Na phosphate buffer, 5 mM K_4_[Fe(CN)_6_] 3H_2_O, 5 mM K3[Fe(CN)6], 150 mM sodium chloride, 2 mM magnesium chloride and 1 mg ml − 1 X-gal in distilled water). The nuclei were counterstained with DAPI. The cells were viewed by bright field microscopy and 10 random fields of view were counted for each sample. In addition, representative kidney sections were embedded in Tissue-Tek® O.C.T. compound (Sakura, Zoeterwoude, Holland), flash frozen in liquid nitrogen and sectioned. Cryosections were fixed with 1% formaldehyde and stained with the same X-GAL staining solution at 37°C over night [44].

### Quantitative Reverse Transcriptase PCR

RNA was isolated from frozen kidney tissue and cultured cells using either the RNeasy Mini kit (Qiagen) or the RNeasy Micro kit (Qiagen) according to the manufactures instructions. Reverse transcription was performed with M-MLV-Reverse Transcriptase (Promega, Madison, US) and random primers. Amplified cDNA was used as a template for qPCR. The levels of NGAL and KIM-1 mRNA expression were determined by quantitative real-time PCR using a Roche Lightcycler 480 System with SYBR green master mix and specific primers: NGAL for: TGA AGG AAC GTT TCA CCC GCT TTG, NGAL rev: ACA GGA AAG ATG GAG TGG CAG ACA; KIM-1 for: AAA CCA GAG ATT CCC ACA CG, KIM-1 rev: GTC GTG GGT CTT CCT GTA GC. Melting curves were examined to verify that a single product was amplified. For quantitative analysis, relative mRNA levels were calculated according to the 2^-ΔΔCt^ method; all samples were normalized to actin gene expression. The levels of p15*^INK4b^*, p16*^INK4a^* and p19*^ARF^*, HPRT mRNA expression were determined by quantitative real-time PCR using an ABI PRISM 7700 Sequence Detector (Applied Biosystems, Foster City, US) with TaqMan® Universal Master Mix and specific primers and FAM-labeled probes: p15*^INK4b^* TaqMan® Gene Expression Assay (Applied Biosystems Mm00483241_m1), p16*^INK4a^* forward: GGG CAC TGC TGG AAG CC, p16*^INK4a^* reverse: AAC GTT GCC CAT CAT CAT C, p16*^INK4a^* probe: CCG AAC TCT TTC GGT CGT A, p19*^ARF^* forward: TCG TGA ACA TCT TGT TGA GGC TA, p19*^ARF^* reverse: GTT GCC CAT CAT CAT CAT CAC CTG, p19*^ARF^* probe: CGG TGC GGC CCT CTT CTC AAG ATC, HPRT for: TGA CAC TGG TAA AAC AAT GCA AAC T, HPRT rev: AAC AAA GTC TGG CCT GTA TCC AA, HPRT probe: TCC ACC AGC AAG CTT GCA ACC TTA ACC. For quantitative analysis, relative mRNA levels were calculated according to the 2^-ΔΔCt^ method; all samples were normalized to HPRT gene expression.

### Immunoblot

Western analysis was performed as previously described [2]. In short, a representative section of the whole kidney was taken, frozen in liquid nitrogen and subsequently homogenized by automated homogenizer. Proteins were ran through gel electrophoresis and blotted onto a PVDF membrane. After blocking, membranes were incubated overnight at 4°C with primary antibodies: anti-LaminB1 (Cell Signaling, 9087), anti-p53 (Cell Signaling, 2524), anti-p21 (BD, 556431), anti-p16 (Santa Cruz, SC-1207), anti-Cyclin D1 (Thermo Fisher Scientific, RM-9104), anti-β-actin (Abcam, ab82618), anti-P-p42/44 (Cell Signaling, D13.14.4E), anti-p42/44 (Cell Signaling, 137F5), and anti-GAPDH (Sigma, G9545). After incubation, membranes were washed and incubated in HRP-conjugated secondary antibodies: anti-rabbit (Cell Signaling) or anti-mouse (Cell Signaling). Proteins were visualized by Supersignal® West Pico Chemiluminescent Substrate (Thermo Fisher Scientific).

### Statistics

Data are shown in mean ± SEM. Statistic significance among multiple groups was determined by two-way ANOVA tests, with a *post hoc* Bonferroni test to determine significance between groups. To determine significance for comparisons between two groups a student's t-test was used. Correlations were performed as Pearson's correlation. *P*<0.05 was accepted as statistical significance. Real-time PCR relative quantitations were determined by the 2^−ΔΔCt^ method [45]. Prism 4.0 (GraphPad Software, San Diego, CA) and Microsoft Excel were used to perform statistical test.
